# Video Consultations Between Patients and Clinicians in Diabetes, Cancer, and Heart Failure Services: Linguistic Ethnographic Study of Video-Mediated Interaction

**DOI:** 10.2196/18378

**Published:** 2020-05-11

**Authors:** Sara E Shaw, Lucas Martinus Seuren, Joseph Wherton, Deborah Cameron, Christine A'Court, Shanti Vijayaraghavan, Joanne Morris, Satyajit Bhattacharya, Trisha Greenhalgh

**Affiliations:** 1 Nuffield Department of Primary Care Health Sciences University of Oxford Oxford United Kingdom; 2 Faculty of Linguistics University of Oxford Oxford United Kingdom; 3 Barts Health NHS Trust London United Kingdom

**Keywords:** delivery of health care, physical examination, remote consultation, telemedicine, health communication, language, nonverbal communication, mobile phone

## Abstract

**Background:**

Video-mediated clinical consultations offer potential benefits over conventional face-to-face in terms of access, convenience, and sometimes cost. The improved technical quality and dependability of video-mediated consultations has opened up the possibility for more widespread use. However, questions remain regarding clinical quality and safety. Video-mediated consultations are sometimes criticized for being not as good as face-to-face, but there has been little previous in-depth research on their interactional dynamics, and no agreement on what a good video consultation looks like.

**Objective:**

Using conversation analysis, this study aimed to identify and analyze the communication strategies through which video-mediated consultations are accomplished and to produce recommendations for patients and clinicians to improve the communicative quality of such consultations.

**Methods:**

We conducted an in-depth analysis of the clinician-patient interaction in a sample of video-mediated consultations and a comparison sample of face-to-face consultations drawn from 4 clinical settings across 2 trusts (1 community and 1 acute care) in the UK National Health Service. The video dataset consisted of 37 recordings of video-mediated consultations (with diabetes, antenatal diabetes, cancer, and heart failure patients), 28 matched audio recordings of face-to-face consultations, and fieldnotes from before and after each consultation. We also conducted 37 interviews with staff and 26 interviews with patients. Using linguistic ethnography (combining analysis of communication with an appreciation of the context in which it takes place), we examined in detail how video interaction was mediated by 2 software platforms (Skype and FaceTime).

**Results:**

Patients had been selected by their clinician as *appropriate* for video-mediated consultation. Most consultations in our sample were technically and clinically unproblematic. However, we identified 3 interactional challenges: (1) opening the video consultation, (2) dealing with disruption to conversational flow (eg, technical issues with audio and/or video), and (3) conducting an examination. Operational and technological issues were the exception rather than the norm. In all but 1 case, both clinicians and patients (deliberately or intuitively) used established communication strategies to successfully negotiate these challenges. Remote physical examinations required the patient (and, in some cases, a relative) to simultaneously follow instructions and manipulate technology (eg, camera) to make it possible for the clinician to see and hear adequately.

**Conclusions:**

A remote video link alters how patients and clinicians interact and may adversely affect the flow of conversation. However, our data suggest that when such problems occur, clinicians and patients can work collaboratively to find ways to overcome them. There is potential for a limited physical examination to be undertaken remotely with some patients and in some conditions, but this appears to need complex interactional work by the patient and/or their relatives. We offer preliminary guidance for patients and clinicians on what is and is not feasible when consulting via a video link.

**International Registered Report Identifier (IRRID):**

RR2-10.2196/10913

## Introduction

### Background

There is a significant push from decision makers across the world to make better use of digital technologies, including video consultations [1-3]. Virtual media such as Skype (Microsoft Corporation), FaceTime (Apple Inc), and Attend Anywhere (Attend Anywhere) are increasingly being used for web-based communication between patients and clinicians. In outpatient departments, drivers include addressing high nonattendance rates and rising costs. Patients are often keen to use video consultations given the potential for reduced time and travel, especially for tertiary care [4-6]. The coronavirus disease 2019 (COVID-19) pandemic has provided significant additional pressure to deliver video-mediated services, rapidly and at scale, to reduce face-to-face social contact and help to reduce the spread of the disease.

Published research on video outpatient consultations has been summarized in several recent reviews [7-10]. These show great potential for video consultations in terms of acceptability, safety, and effectiveness in patients considered clinically eligible across a range of conditions, such as diabetes [11-15], ophthalmology [16], cancer [17,18], chronic kidney disease [19], spinal cord injury [6,20,21], chronic obstructive pulmonary disease [22,23], mental health conditions [24,25], Down syndrome [26], cerebral palsy [27], chronic pain [18,28,29], therapies (eg, speech and language therapy [30-32]), support after premature birth [33], support of patients in care homes [34], and plastic surgery [35].

Much of this literature uses either experimental methods, classifying service models primarily by the technology used and secondarily by the task or clinical work supported by that technology, or questionnaires and semistructured interviews, typically with small samples, to assess feasibility and acceptability. There is limited evidence on costs [36]. Patients have expressed some concerns about privacy and security and ease of use of the technology [37,38]. Some studies have reported less enthusiasm for video consultations among older people [39,40]. Qualitative studies have reported concerns over technical issues such as pixilation or audio quality and quality of care, for instance, because of the lack of physical examination [9,38,41,42].

There has been limited research focused specifically on the dynamics of video-mediated interaction in health care consultations and the ways in which communication might be altered by the use of video. To date, only 8 studies have examined such interaction in video consultations, with only one analyzing video recordings at both *ends* of the consultation. Ekberg et al [30,43] looked at the use of virtual media to support cognitive behavioral therapy and speech and language therapy, finding that in both cases, practitioners were able to adapt consultations and communication accordingly. In a series of studies on video-mediated vascular and cardiology consultations, Pappas et al [44-46] explored video consultations in which the patient, supported by a general practitioner (GP) or nurse, consulted remotely with a specialist from their GP practice. They found that clinical assessment and decision making worked well and that the video medium supported collaborative discussion and shared care. However, professionals needed to adapt their interaction to accommodate the video medium (eg, switching between consultative and interprofessional talk, while manipulating camera angle) so as to ensure ongoing patient involvement [44-46]. A previous study by our team compared video consultations of diabetic and postoperative cancer patients with matched face-to-face consultations and provided an overview of what is gained and what is lost when clinicians and patients interact remotely for instance, finding that the overall length of a video consultation is shorter and there are more breaks in the conversation compared with an equivalent face-to-face encounter [9,47]. Stommel et al [48] studied openings of postsurgical video consultations, revealing small but significant interactional differences, with more prosocial talk at the start of video consultations, and the role of companions in a video consultation, revealing that they are mostly off-screen, invisible to the clinician, and rarely involved in the interaction [49].

There is a growing body of evidence outside of health care that highlights the ways in which video calling might alter interaction [50]. Early studies on video-mediated interaction highlighted how technology could be detrimental to conversational flow [51-53]. In face-to-face interaction, people can see and hear actions as they are produced, but in video-mediated interaction, they see and hear them milliseconds later, a phenomenon known as latency. Such delays seem small but are meaningful in conversation as they interfere with the system of rules that participants use to determine who gets to talk at which point in the conversation (turn-taking [54]) and address problems such as misunderstandings (repair [55]). By preventing participants from adequately applying these rules, video technology can affect the quality of interaction and user experience. Other studies raise questions about if technical failures, such as hearing no sound or new types of interruption, for instance, a family member entering the room, impact the interaction [56-58]. Such evidence has yet to be considered in relation to video consultations in health care.

In summary, although the evidence on video consultations indicates that they are feasible, safe, and effective in health care, the evidence on interaction in such consultations is limited. Only a handful of studies have considered the impact of the video medium on the microdynamics of interaction in medical consultations [36]. The result is that little is currently known about how different communication strategies, types of communication (speech, bodily conduct, gaze, and posture), the material properties of technology, and/or the quality of web-based connection shape interaction in video consultations in health care.

We studied interaction in video consultations in four clinics in two National Health Service (NHS) trusts (one acute and one community). The Qualitative Analysis of Remote Consultations (QuARC) study built on several years’ work by our team looking at the national-level context for video consultations, the organizational context supporting adoption and implementation, and microlevel interactions between patients and clinicians [9,47,59]. The QuARC study combined data from 2 previous studies—the Virtual Online Consultations-Advantages and Limitations (VOCAL) study, a National Institute for Health Research (NIHR)–funded study of diabetes and cancer clinics in East London using Skype, and the Oxford Telehealth Qualitative Study (OTQS), a Wellcome Trust-funded study of specialist community heart failure teams in Oxford using Skype or FaceTime—and conducted detailed interactional microanalysis across video consultation data.

### Aims, Objectives, and Research Questions

As set out in the QuARC study protocol [10], the aims of the study were to identify and analyze the communication strategies through which remote consultations are accomplished and to produce guidance for patients and clinicians to improve the communicative quality of video consultations. Specifically, our objectives were (1) to conduct a secondary analysis of a multimodal dataset of 37 remote consultations with diabetes, antenatal diabetes, cancer, and heart failure patients and their clinicians using a combination of ethnographic and microanalytic approaches to investigate how interaction is affected by mediation via Skype or similar apps and (2) develop guidance for patients and clinicians in conducting remote consultations.

Our research questions were as follows:

1. What are the (often implicit or unspoken) communication strategies through which technology-mediated consultations for diabetes, cancer, and heart disease are successfully accomplished?
2. How do patients and clinicians negotiate misunderstandings in technology-mediated consultations, and what strategies are more effective?
3. What can we learn from a detailed linguistic analysis of real-life remote consultations to guide other clinicians and patients interested in or actively using Skype and other video platforms for medical consultations?

## Methods

We have previously published a detailed study protocol [10]. This section provides a summary along with an update on the analytic approach.

### Study Design and Approach

A mixed methods design was used to study video consultations in 4 contrasting clinical settings (diabetes, antenatal diabetes, cancer, and heart failure patients) across 2 NHS trusts (Oxford and London). The study was informed by linguistic ethnography, which uses both linguistic and ethnographic approaches to understand how social and communicative processes operate in a range of settings and contexts, combining video and audio recordings of consultations, interviews, and observations from 2 previous studies (VOCAL and OTQS, detailed above) to examine in detail how patient-clinician interaction is shaped by use of Skype and FaceTime.

### Project Management and Governance

This study was delivered by a core working group (SS, LS, DC, JW, and TG) and supported by an independent steering group with a lay chairperson and cross-sector stakeholder representation, including patients, NHS stakeholders, and national-level decision makers.

Both studies from which data were drawn received ethical approval for a detailed analysis of video recordings of video consultations. VOCAL was approved by the National Research Ethics Service Committee London–City Road and Hampstead in December 2014 (14/LO/1883) and OQTS by the South Central–Berkshire Research Ethics Committee in September 2015 (15/SC/0553). All participating staff and patients in both studies gave their informed consent to be audio and video recorded during consultations and for the data to be used for research purposes.

### Setting

The study took place at 2 NHS trusts in London and Oxford, each of which explored the use of video consultation services. Table 1 provides an overview of the clinical settings in which recordings of consultations took place. The diabetes service in London had an established video consultation service, whereas the other 3 were piloting or setting up video consultations at the time of the respective studies. Video consultation services in heart failure and antenatal diabetes ceased at the end of the respective studies. Further details on each of the settings can be found in publications from VOCAL [9,47] and OTQS (C Papoutsi et al, unpublished data, 2020).

**Table 1:** Summary of the four clinical settings in which video consultations took place.

Clinical setting	Population	Clinical provision	Staff	Video consultation service
Diabetes services (London)	Adult/young; adult patients (18-80+ years), with high prevalence of type 2 diabetes in ages 16 to 25 years, plus significant risk factors (eg, poverty, diet, or ethnicity)	Integrated community diabetes service, with consultants providing 6-monthly reviews and ongoing support from diabetes nurse specialists	Lead diabetologist, 5 consultant diabetologists, and 6 specialist nurses	Established in 2010 because of typically low engagement with traditional service models, poor health outcomes, increasing use of unplanned care via A&E^a^; delivered largely by lead diabetologist (who offered virtual consultations to all adult/young adult patients as an alternative to follow-ups), with other staff slowly coming on board; using Skype (consumer version) on desktop at the time of the study
Antenatal diabetes services (London)	Expectant mothers (around 350 per year) with gestational diabetes	Outpatient consultations (including preappointment tests and checks) combined with optional weekly telephone clinic (for those needing close monitoring); key medical information (eg, blood sugar readings) stored in patient-held maternity folder	3 diabetes consultants, 3 obstetricians, 2 nurses, and 1 midwife	Piloted as part of the VOCAL^b^ study, with video consultations led by 1 consultant and using Skype (consumer version) on a clinic desktop
Hepatobiliary and pancreatic cancer surgery services (London)	Patients with pancreatic/liver cancer who had major surgery and a prolonged postoperative phase; diverse demographic, living up to 200 miles from clinic	Tertiary service, with clinic run once per week, 2 to 3 patients were typically seen for postoperative cancer follow-up	1 consultant surgeon, 2 specialist registrars, 1 clinical nurse specialist, and nurse assistants	At the start of the VOCAL study, the clinic had begun to introduce virtual consultations to spare selected patients unnecessary travel, run in a shared hospital space alongside other clinical services, and using Skype (consumer version) on a clinic desktop
Heart failure service (Oxford)	Heart failure patients (typically 65+ years) with reduced ejection fraction, many unable to get to clinic (owing to frailty or severe symptoms)	Community outreach service delivered by heart failure specialist nurses working with the hospital-based heart failure service, local general practitioners, other community services, social services, and ambulatory assessment units	5 specialist heart failure nurses	Piloted at the time of the OTQS^c^ study to evaluate if video consultations could help deploy limited resources safely, efficiently, and effectively without loss of patient or staff satisfaction. Heart failure specialist nurses were equipped by their employing trust with iPads with SIM cards to enable real-time access to patients’ records, enabling the use of Skype or FaceTime

^a^A&E: accident and emergency.

^b^VOCAL: Virtual Online Consultations-Advantages and Limitations.

^c^OTQS: Oxford Telehealth Qualitative Study.

### Sampling and Data Collection

We collected data over 30 months (VOCAL, 2015-2017; OTQS 2016-2017). The data sources are summarized in Table 2, with further details below.

Our dataset consisted of 37 video recordings of consultations (Table 2) from the VOCAL and OTQS studies. The goal of sampling in both studies was to capture the breadth of experience of video consultations, seeking maximum variety in clinical, social, ethnic, and personal circumstances (Table 3) and in health and information technology literacy. It was a precondition of ethical approval that clinicians could exercise judgment about which patients to invite to the study.

Exclusion criteria were no 3G internet access at home, lack of familiarity with the technology, clinical inappropriateness (eg, in London, the need for direct physical examination), inability to give informed consent, and comorbidity preventing participation (eg, severe visual impairment). In the diabetes clinic, which included a high proportion of limited English speakers, bilingual health advocates trained in the use of remote consulting were available, so limited English was not an exclusion criterion there. In the antenatal diabetes and cancer surgery clinics, those who were comfortable with a family member interpreter were included, but a remote interpreting service was not available. In the heart failure service, all participating patients were native English speakers.

To enable comparison, we collected 28 audio recordings of matched face-to-face consultations (Table 2). Participants were selected on the basis that the type of consultation was similar to those conducted remotely (eg, routine follow-up cancer appointments) and that the consultant would consider such cases suitable for a video consultation.

**Table 2:** Overview of data and analysis of the Qualitative Analysis of Remote Consultations study.

Type of data	Data description	First order interpretation	Higher order interpretation
Consultation data	Video recordings and screen capture (at patient end and clinician end) of 37 virtual consultations (12 diabetes, 6 antenatal diabetes, 12 cancer, and 7 heart failure); audio recording of 28 face-to-face consultations (7 diabetes, 6 antenatal diabetes, 6 cancer, and 9 heart failure)	What is said and done in consultations (video and face-to-face); unfolding interaction and strategies for communication; how technology is used in consultations (video and face-to-face); and how participants felt	How people interact and communicate, how people create and maintain order and coherence in consultations together, and how video technology shapes, enables and constrains this; the relevance of different channels (verbal, visual, gesture, or gaze); and how these all shape the actions of users
Contextual data	Accounts of 26 patients before/after the appointment (19 from VOCAL^a^ and 7 from OTQS^b^) and 35 staff involved in delivering video consultations (28 from VOCAL and 7 from OTQS) combined with field notes from before/after face-to-face and video consultations at patient and clinician end Documents (16 from VOCAL; 7 from OTQS) (eg, operating procedures and meeting minutes) Researcher field notes about people and technologies delivering video consultations; including diagrams of how people, technologies, and clinical work interact Demographic data	Key interactions and interdependencies; key organizational routines and how these are changing over time; and accounts of clinical work and how this is shaped or reshaped through use of video consultations Basic patient information, including age, gender, and ethnicity	Social structures (eg, professional standards and definitions of excellence; what actors *know* and how they interpret the strategic terrain) and assumptions built into the technology about, for example, capability of users, how people interact, privacy and consent, the nature of clinical work, and how all these interact Background and context to detailed micro-analysis

^a^VOCAL: Virtual Online Consultations-Advantages and Limitations.

^b^OTQS: Oxford Telehealth Qualitative Study.

**Table 3:** Overview of consultations in the Qualitative Analysis of Remote Consultations dataset.

Clinic	Total recorded	Male or female	Age (years), median (range)	Ethnicity
Diabetes (video)	12	5 male, 7 female	23 (21-50)	White British (5); white other (2); black Caribbean (1); Asian Bangladeshi (1); Asian Indian (3)
Diabetes (face-to-face)	6	3 male, 3 female	26 (21-58)	White British (2); black Caribbean (1); Asian Bangladeshi (2); Asian other (1)
Antenatal diabetes (video)	6	6 female	34 (30-37)	White British (1); black Caribbean (1); Asian Bangladeshi (1); Asian other (3)
Antenatal diabetes (face-to-face)	6	6 female	33 (26-36)	White British (0); black Caribbean (1); Asian Bangladeshi (3); Asian Indian (1); Asian other (1)
Cancer (video)	12	4 male, 8 female	74 (55-85)	White British (9); white other (1); black Caribbean (1); Asian Indian (1)
Cancer (face-to-face)	5	3 male, 2 female	69 (45-75)	White British (2); black Caribbean (2); Asian other (1)
Heart failure (video)	7	3 male, 4 female	67 (33-87)	White British (7)
Heart failure (face-to-face)	9	1 female, 8 male	60 (56-78)	White British (9)

Video consultations for the VOCAL study were recorded by a researcher (JW) using a small digital camcorder with a wide-angle lens. For the OTQS study, they were recorded by a researcher (CA/TG) using a small digital camcorder and a handheld iPad (Apple Inc). Face-to-face consultations were audio recorded using a digital Dictaphone. We captured clinician and patient interaction with the videoconferencing software using a commercially available screen-capture software tool (ACA Systems) to record screen images showing on each party’s computer screen as a video file. This was run directly from a USB memory stick.

Each end of the consultation resulted in 2 digital files: 1 screen capture and 1 video. We synchronized these into 1 file using video editing software, meaning that the video of the computer screen could be played exactly in parallel with a video of the patient looking at the screen, and then aligned the patient and clinician *ends* (an example screenshot is shown in Multimedia Appendix 1). In several cases (Table 4), the consultation was recorded only at one end. This was either because the patient did not wish to be filmed in their home but was happy to be filmed from the clinic or because it was not practically possible to arrange for recording to take place at both ends.

We transcribed all face-to-face and video consultations using a specialized program, Transana [60], which allows researchers to capture the complexity of data, such as body language, while simultaneously following video, audio, and transcription (Multimedia Appendix 1).

In both studies, we made contemporaneous field notes at each patient’s home and at the clinic, for instance, relating to the patient’s material circumstances or the physical circumstances in which the clinician makes the remote call. We interviewed key staff at each site involved in setting up and conducting video consultations and patients as soon as possible after their video consultation.

**Table 4:** Summary of video consultation recordings.

Video recording	Cancer	Diabetes	Antenatal diabetes	Heart failure	Total
Dual clinic, dual home	4	0	1	2	7
Dual clinic, single home	1	0	1	3	5
Single clinic, dual home	1	2	1	1	5
Single clinic, single home	3	1	3	1	8
Dual clinic, no home	2	7	0	0	9
Single clinic, no home	1	2	0	0	3
Total	12	12	6	7	37

### Theoretical Approach

We used 2 complementary theoretical approaches that see communication as a dynamic interaction that emerges moment by moment, which allowed us to examine the role of technology in shaping interaction. First, we used the *ethnography of communication* [61] to produce systematic and richly contextualized descriptions of the communicative genres, events, and practices that are observed in a particular culture to identify the key features of video consultations and attend systematically to contextual factors (eg, lack of spatial proximity and restricted visual field) that may produce differences with face-to-face consultations. Second, we used conversation analysis (CA) [62] to guide the fine-grained examination of the patterning of interaction, (ie, how consultations are managed by participants moment by moment). We focused on the linguistic, bodily, and contextual resources used by participants to create and maintain order and coherence and how different modes and channels of communication, such as the verbal and visual, shape actions (eg, physical examination).

### Analysis

As is usual in linguistic ethnography, the analysis combined *zooming in* on interaction to understand how video consultations are successfully conducted, with *zooming out* to the clinic and wider organization to understand the context in which they take place. This process began with repeated viewings of video recordings among the team, which included representation from linguistics, sociology, psychology, and medicine with the aim of identifying “recurrent and stable details of talk-in-interaction” [62]. From this, we identified 4 questions for an in-depth study:

1. How do patients and clinicians open and close video consultations?
2. How are physical examinations accomplished in video consultations?
3. How do patients and clinicians address problems with the technology?
4. How is turn-taking affected by the video-mediated setting?

For the first 2 questions, we built collections of all openings, closings, and physical examinations, respectively. For the third question, we built a collection of cases in which the technology failed (eg, there was no audio) or where a failure in the technology was addressed (eg, a patient says his image is frozen). For the fourth question, we built a collection of cases where the patient and clinician struggled with smooth turn-taking, and so had repeated overlapping talk and/or stopped talking. Silences and overlapping talk are common in all conversations [54]; hence, not all cases of overlap and silence in our data were necessarily a result of the video medium. We therefore (1) only included consultations recorded at both patient and clinic ends, allowing us to determine that latency (ie, the transmission delay between one participant saying something and the other hearing it) was an issue, and (2) focused on points in the conversation where speaker transition should take place, but the latency caused problems.

In line with CA [63,64], we transcribed data using Jeffersonian and Mondada systems (Multimedia Appendix 2), employing a range of symbols and conventions to capture *what* participants say, *how* they say it (eg, pauses and volume), and accompanying *nonverbal* communication (eg, gaze and head movement). Guided by the principles of sequence organization and turn design [65,66], we then examined each case (eg, individual opening) to determine the actions that participants performed, how these actions were organized in the consultation [67,68], and the communication practices that participants used to make these actions recognizable. We then searched for patterns and commonalities across each collection to distinguish between communication strategies that were relevant for single instances and those used more systematically, for instance, the ways in which clinicians use different opening questions. In other words, we identified the norms and conventions that participants appeared to rely on in video consultations.

Finally, once we had a solid appreciation of the patterns of communication across collections, we identified areas where there was *trouble*, such as potential misunderstandings, and grouped these into key challenges. At this point, we *zoomed out* to our contextual data with the aim of better understanding how and why such challenges occur (eg, the way in which Skype technology is set up on computers and what this requires of clinicians when they open video consultation) and how they are negotiated.

### Patient and Public Involvement

Patients and their caregivers were key to the research. We set up a dedicated patient advisory group (PAG) in 2015 to enable patients to review documents and give feedback on their experiences and to incorporate patient feedback within the respective studies. We have since set up a standing group, Patients Active In Research on Digital health, who, along with the PAG, have reviewed emerging findings (including anonymized video clips) from the QuARC study and provided feedback on developing guidance on video consultations. Finally, we worked with a design company to co-design guidance and an accompanying animation that involved 2 workshops with a total of 50 members of the public, followed by a review of materials via email. This process was critical in developing both content and design of these outputs (eg, guiding us not only to focus on how to do a video consultation but also to include technical setup).

## Results

### Overview of Findings

We found that for this highly selective group of patients and clinicians, the use of video technology typically (but not always) enabled effective consultations that followed the conventional organization of a consultation [68]. Patients and clinicians adapted quickly and easily to the video-mediated context, transferring communication strategies from face-to-face communication to successfully complete web-based consultations. For example, in video consultations, the webcam is positioned above the screen, making mutual gaze impossible, but participants had no problem positioning their head and shoulders in front of the camera and looking at the screen to show that they were engaged and directing their attention to their coparticipant.

Clinicians managed to start consultations in the same way as a face-to-face consultation by asking, for instance, “How are you today?” and inviting patients to provide updates on their condition since the last consultation [69]. Patients responded in the conventional way, either reporting no problems or highlighting recent changes (eg, high blood sugars). When clinicians were engaged in other activities, such as updating the patient’s record, they made this clear by saying, for instance, “If I go silent, I’m writing. Okay?” Closing of consultations was straightforward, and followed the same pattern as face-to-face consultations, with the clinician checking if the patient had any further questions and then closing down the discussion, arranging a follow-up as needed, and saying goodbye.

The use of video calling (30 Skype, 7 FaceTime) was a novel form of communication for many users (particularly for older participants, Table 3). Younger patients appeared particularly comfortable with the technology, but even those with less technological affinity had few problems using the technology once they had some assistance, for instance, from a carer. When clinicians and users initially made contact, we observed that in many cases, consultations were characterized by surprise, amusement, and a sense of informality. This often continued into the consultation, with participants sometimes joking, generally, and about the use of video. It was striking that those clinicians who were new to the video medium felt quite distracted by having their own image appear on the screen for the first time—working out how to minimize or ignore it was an important step in re-establishing a formal, professional focus.

It was when we began to look at these issues in detail that we became interested in how it is that people interact effectively via video. Across the 37 video consultations in our dataset (12 hours and 21 min of total consultation time), we identified 3 key challenges that (some but not all) participants faced when doing video consultations: (1) establishing a connection and starting a video consultation, (2) dealing with disruption to conversational flow caused by technical issues with audio or video, and (3) conducting a physical examination via video. We describe each of these below, along with the communication strategies used to negotiate them. We present data extracts illustrating the analysis in the figures below (full transcriptions following CA conventions are included in Multimedia Appendix 3).

### Challenge 1: Establishing a Connection and Starting a Video Consultation.

The start of a video consultation is a crucial time when the patient and clinician seek to establish a technical connection and determine if this is good enough for the consultation to go ahead. This process was not always straightforward. The combination of consultation and contextual data in our study enabled us to distinguish between *preopenings* involving steps to establish a connection and *openings* involving greetings like *hello*, followed by *how are you?* type questions [69] that signal the start of the clinical consultation.

Before a video consultation can get started, steps must be taken at both *ends* to enable the patient and clinician to establish a connection. For some participants, this opening sequence could be stressful, as they sought to ensure that the relevant equipment was in place and the technical connection was working to enable the clinical element of the consultation to begin. Depending on the technology, participants needed to first identify each other via their web-based user name (ie, exchanging remote addresses) and exchange phone numbers and/or email addresses (also, at the clinic end, ensure that internal procedures, standards, software functionality, and administrative systems are in place to support the consultation; refer to Greenhalgh et al [70] for further details). The clinician would then usually *dial* the patient, who, by answering, would show that they were ready for the consultation. On the whole, preopenings ran smoothly. In some cases, participants experienced trouble, including outdated software that required *on the spot* updates (n=2), lost passwords (n=2), or misunderstandings about which platform to use (n=1). The problem was typically resolved by one participant (usually the clinician) calling the other by telephone, finding a solution, and then restarting the video connection.

Once a connection was established, opening greetings at the start of a video consultation were critical, not only in prompting conversation but also in indicating that participants could see and hear one another. It was at this point when technological issues, such as limited audio, were acknowledged and addressed. When clinicians and patients were familiar with the technology and potential problems, openings ran smoothly, and problems were quickly resolved. However, when one or more participants were not familiar with the technology, this could quickly lead to confusion and delay (often related to issues of breakdowns and latency, discussed below).

**Figure 1:**
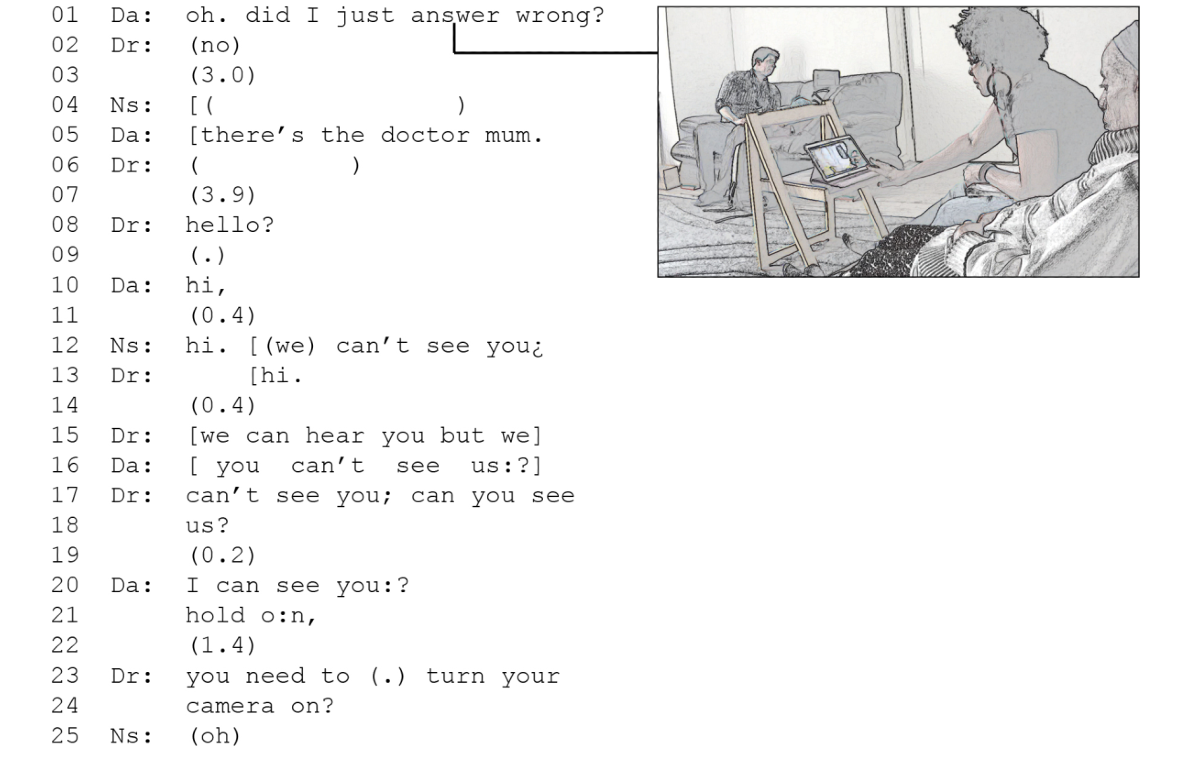
Example of a clinician and patient establishing a connection at the start of a Skype consultation. Da: daughter; Dr: doctor; Ns: nurse.

Take the example in Figure 1, which was taken from the start of a postoperative cancer consultation in which the patient’s relative did not turn on the camera when she answered the doctor’s video call; in the middle of line 1, when the daughter says *answer*, the doctor appears on their screen (screenshot, Figure 1).

In this example, the patient and clinician have already done the work of preopening, the patient has been invited to a video consultation, with Skype addresses and video appointment times confirmed to enable that to happen, and phone numbers confirmed ensuring an alternative means of communicating. Once a connection is established, the patient and clinician do not greet each other but instead query the connection and confirm at the patient’s end that they can see the doctor. Then, 2 periods of silence follow (3.0 and 3.9 seconds, respectively). During this time, the doctor is unsure what is happening: by *not* greeting, the patient and her daughter do not provide the doctor with evidence that they have a working video connection meaning that, for the doctor and nurse at the other end, it is not clear if there is a problem with the connection or video. The doctor’s *hello* with its strong rising pitch (line 8) is not a greeting, but a means of testing if the patient and/or daughter can hear him—a practice that we found repeatedly when there was a problem with the connection and participants wanted to see if they could still hear each other. When the daughter is heard to have responded with *hi* (line 10), this provides evidence that they can hear, that the connection works, and that the problem is purely visual (as the nurse points out in line 12). The daughter moves to turn the camera on, the doctor confirms that they can now see, and the consultation begins. Unlike in a face-to-face consultation, it is only at this point that the doctor asks *how are you*, signaling the start of the clinical consultation and enabling the social rituals of *being a doctor* and *being a patient* to begin.

### Challenge 2: Dealing With Disruption to Conversational Flow

The technology used in video consultations is intended to facilitate interaction. In our dataset, there were only 10 (of 37) consultations in which participants experienced no problems with the audio or video. In the other 27 consultations, we identified 49 instances in which a breakdown in audio and/or video disrupted the conversational flow. In 25 of these consultations, we had recordings at both *ends* (ie, clinician and patient; Table 3), allowing us to identify 151 cases in which latency disrupted interaction.

Such disruptions are significant as they not only disrupt conversational flow but also potentially impact the quality and outcome of consultations (Table 5). We provide a detailed analysis of the breakdowns and latency below.

**Table 5:** Frequency and duration of breakdowns and latency issues in video consultations.

Type of problem			Frequency^a^		Duration (range)	
**Audio or video breakdown at the start of a consultation**						
	**No sound at the start**					
		Unsolved: clinician calls the patient on the phone using Skype for video	3		6.5 to 9.5 min	
		Solved: after disconnecting and reconnecting, the sound works	2		53 to 127 seconds	
	**No video at the start**					
		Unsolved: camera does not work and participants make do with audio only	1		5 min and 5 seconds	
		Solved: either participant had forgotten to turn on the camera	8		6.8 to 22.3 seconds	
**Audio or video breakdown during a video consultation**						
	Garbled sound: the quality of the sound suddenly degrades, causing a problem with audibility, the consultation is halted, participants check when the audio works, and then resume the consultation			24		3.7 to 56.8 seconds
	Bad audio throughout: the quality of the audio is poor because of technical problems, causing frequent noise or low volume			3		Continuous^b^
	Video cutout: the video briefly cuts out on one side, before automatically resuming; may happen because of an incoming call			2		1.4 to 8.4 seconds
	Bad video throughout: the quality of the video is poor because of a bad internet connection, causing the image to blur, freeze, or even cut out completely			1		Continuous^b^
**Breakdown in connection during a video consultation**						
	Automatically solved: the connection cuts out briefly, but resumes automatically; participants briefly discuss and check if the connection works before resuming			2		6.1 to 15.4 seconds
	Requiring reconnection: the connection is dropped completely and participants have to redial to get the connection back			3		43.5 to 71.9 seconds
**Latency**						
	Brief overlap: participants talk at the same time, but either drops out after 1 or 2 syllables of overlapping talk			122		Up to 0.5 seconds
	Competition for turn: participants talk in overlap for a while, using multiple explicit strategies to figure out whose turn it is			29		0.5 to 10.5 seconds

^a^Reporting the number of problems we identified in the dataset relating to latency and breakdowns.

^b^It is not possible to report exact duration as there were problems, either with audio or video, throughout. This results in continuous issues.

#### Breakdowns

Breakdowns in audio and/or video quality occurred at different times in video consultations and with variable duration and impact (Table 5). At the start of consultations, we identified 5 cases with no audio and 9 cases with no video. In the 5 cases with no audio, 3 were in the cancer service (because of technical problems caused by interference with other devices) and involved the clinician taking up to 9.5 min to try to solve the problem before calling the patient by phone and using video calling merely for the visual connection (once the other devices were identified, such interference was resolved), 1 involved the clinician disconnecting and reconnecting twice and using the inbuilt testing service (taking 2 min and 7 seconds), and 1 involved the patient disconnecting and then reconnecting (53 seconds). Audio breakdowns were only observed in Skype consultations.

Problems with the video connection at the start of consultations were typically the result of the patient or clinician not turning on their camera (8 cases). In 1 case, the clinician could not get the camera to work and, after just over 5 min, decided with the patient that they would proceed with an audio-only consultation.

Problems during consultations were often (but not always) disruptive. We found 24 cases where there was a minor problem with the audio resulting in, for instance, soft or garbled talk (Table 5). These lasted up to 57 seconds but were typically resolved in less than 10 seconds, with participants waiting for audio to be restored. The patient or clinician then pointed out that they had not heard something, and the other participant then repeated their last sentence before continuing with the consultation.

**Figure 2:**
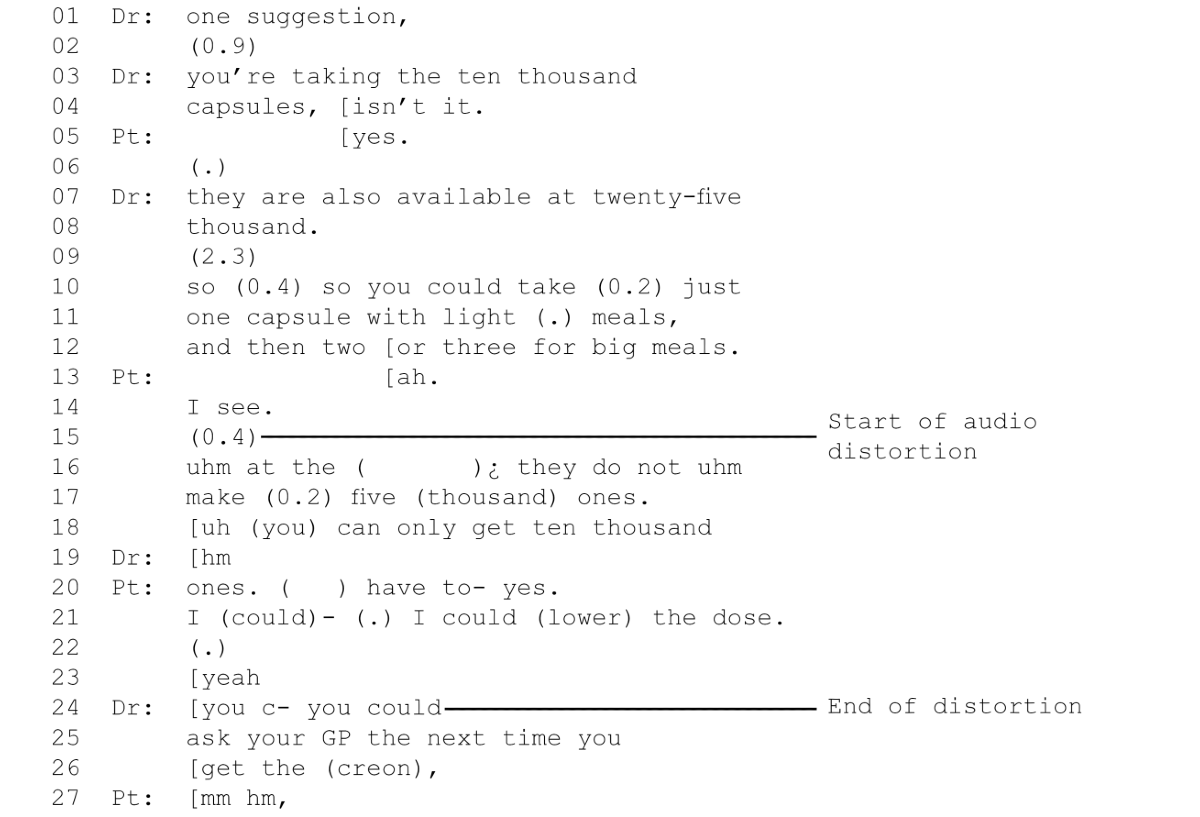
Example of significant disruption to a Skype consultation due to audio problems. Pt: patient.

In 4 consultations, the quality of the hardware or a slow internet connection caused persistent problems with audio (n=3) or video (n=1). In 2 cases, one with a low volume on the patient’s end, the other with a repeated *frozen image* of the patient, participants attempted to solve the problem initially and then agreed to *make do*. In the other 2 cases, the audio frequently suffered from noise or distortions. For 1 consultation, this led to repeated minor problems (n=13), with each party repeatedly unable to hear, and halting the consultation to seek clarification. In the other, a postoperative cancer consultation, there was distortion making it hard to hear, resulting in a misunderstanding about the medication dose. In the extract in Figure 2, the doctor recommends that the patient shift to a higher dose formulation of Creon (pancreatic enzyme replacement)—25,000 instead of 10,000 units; however, the patient mishears and the level of disruption to the audio then limits opportunities for the patient to indicate that they have not understood, what conversation analysts refer to as limited *repair space* [55,71].

In this example, the clinician’s recommendation to change to a 25,000-unit strength capsule is indistinctly audible at the patient’s end, but the patient appears to mishear and to understand that the consultant wants her to start taking 5000-unit strength capsules (line 17). She indicates that she understands with “ah I see” (lines 13-14), but then points out that Creon is not available in 5000-unit strength (lines 16-17), revealing her misunderstanding. The clinician then has the opportunity to rectify the problem [72], but audio distortion, caused at least in part by the microphone at the patient’s end, means that the doctor appears to have trouble making out what the patient is saying and to assume that she heard correctly, recommending that the patient ask her GP for a change in unit strength (lines 24-26). The technical breakdown means that he does not notice that the patient misheard and so continues with the misunderstanding unnoticed and unresolved.

We found 2 cases in which the video was temporarily disrupted on one end of the consultation. In each case, the problem was quickly and automatically resolved (Table 5).

In 5 cases, the connection cut out completely: in 2, the connection was quickly and automatically restored, and in 3, participants had to reconnect themselves.

#### Latency

Spoken conversation is characterized by a turn-taking system, a set of rules participants use to determine whose turn it is to talk. This system serves to minimize both silence and overlapping talk in a conversation, the principle being 1 speaker at a time [54]. Research has shown that participants in a conversation favor direct responses (each person taking over immediately when the other has finished speaking) [73]. Delays in turn-taking and significant overlap usually indicate that there is a communication problem [74]. In our dataset, we found that short periods of latency (up to approximately 200 milliseconds) were tolerated or ignored, but that more significant latency (approximately 500 milliseconds and above) interfered with this system, resulting in silence and overlapping talk or interruption.

The 151 cases of significant latency that we found were unevenly distributed across our dataset, with 67 instances in 21 consultations in which problems were resolved in less than 10 seconds and another 84 instances in only 4 consultations in which problems were not only more frequent but typically took longer to resolve. In 1 of the 4 consultations, there were 47 instances of latency.

Of the 151 cases of latency, 122 were quickly resolved with 1 participant dropping out (ie, stopping talking) after 1 or 2 syllables of overlapping talk (Table 5), and the conversation quickly resumed with minimal impact on flow. In the remaining 29 cases (23 of which were from the 4 consultations with repeated instances of latency), problems were more disruptive and took longer to fix. Here, participants actively competed for the right to talk, resulting in complete sentences overlapping. Latency resulted in longer exchanges in which participants had to work out whose turn it was to talk, which regularly took between 0.5. 10.5 seconds. Conversations were resumed by participants saying something like *sorry* or *go on* and/or repeating the last thing the other person said.

**Figure 3:**
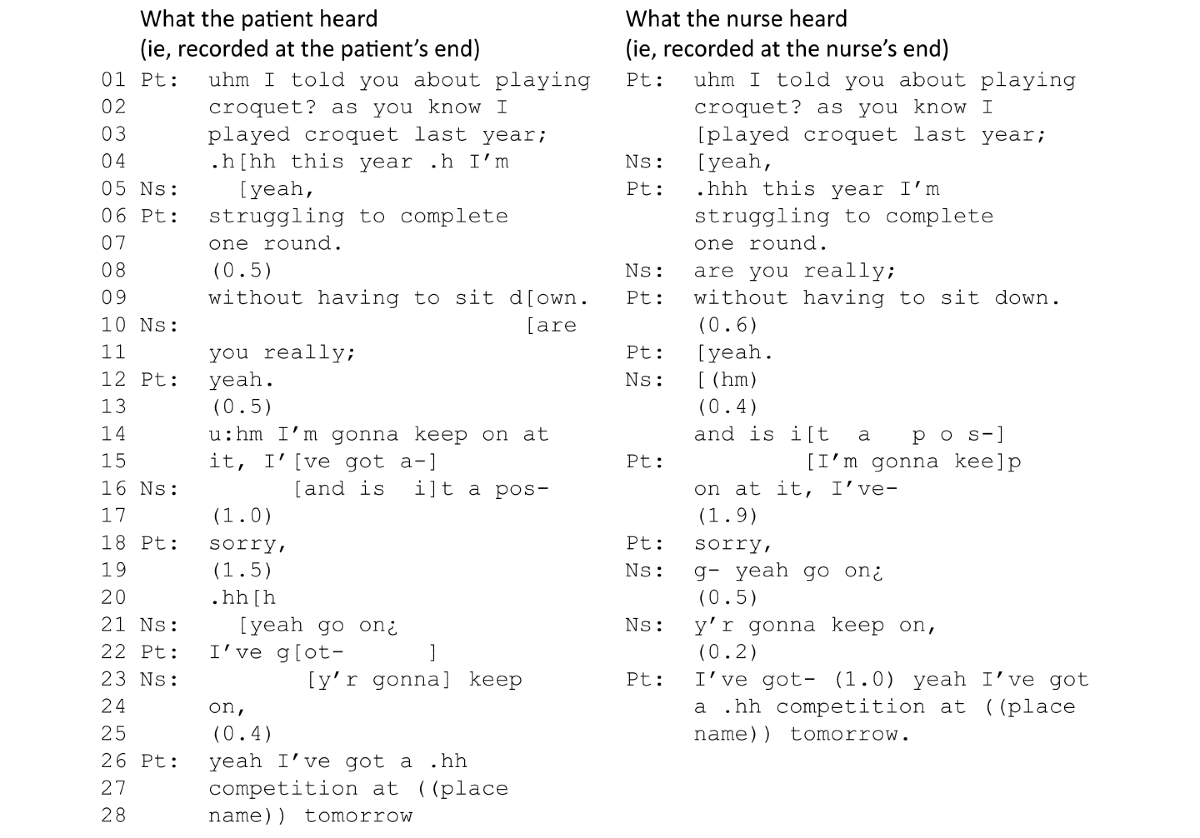
Example of latency disrupting conversational flow in a video consultation for heart failure. Ns: nurse; Pt: patient.

Consider the example in Figure 3, from a heart failure consultation in which the patient is talking to a specialist heart failure nurse about their back problems. Latency in the connection makes the discussion challenging. As the patient tells the nurse about his back problem and the restrictions it places on his day-to-day life (column 1, line 15), the nurse seemingly interrupts to offer a solution (line 16) before the patient has resumed his story. This supposed *interruption* is a result of latency. As can be seen from the nurse’s end (column 2), from her point of view, she does not begin to talk in the middle of the patient’s turn (line 16), but actually starts her turn before the patient (line 15). Due to the latency in the call, she cannot know that the patient has already begun talking again; similarly, the patient cannot realize that the nurse has begun to offer a solution to his presented problem. The result is overlapping talk. Both also deal with different problems: from the patient’s perspective, the nurse interrupts him; from the nurse’s perspective, it is the other way around. However, the latency means that neither of them can know this. Eventually, both then stop talking, and it takes a series of silences and *repair sequences* (eg, the patient’s *sorry*, and the nurse’s *yeah go on*) before the patient can resume his narrative (line 22).

In the 29 cases where latency caused disruptions to conversational flow, participants relied on the same communication strategies they use in face-to-face conversation to try to negotiate the problem. However, latency also caused problems when using these strategies. For example, in Figure 3, the nurse tells the patient to *go on* (line 21), and the patient then resumes his talk as is usual when face-to-face but, because of latency in the connection, the nurse does not realize that the patient has resumed his talk. The nurse then repeats part of his talk (line 23) as an additional strategy to give him the turn, causing the patient to again stop talking (line 22) and confirm before definitively resuming his talk (line 26).

### Challenge 3: Conducting Physical Examinations Via Video

Clinicians and patients are concerned about the appropriateness and safety of conducting physical examinations in a video consultation [75,76]. Data from the heart failure service (including 7 video and 9 face-to-face consultations combined with contextual data about the setting; Table 2) provided a unique opportunity to examine what is possible in terms of physical examination in routine heart failure reviews between specialist heart failure nurses working from a community hospital and patients in their homes. Typically, in a face-to-face consultation, heart failure nurses measure weight, blood pressure, oxygen saturation, and heart rate and rhythm; they also assess edema (fluid build-up in the soft tissues), usually by examining the lower legs, and listening to the patient’s chest with a stethoscope for signs of fluid overload or infection. With the exception of listening to the chest, the same examination can be attempted remotely, using a blood pressure monitor put on by the patient or relative (perhaps incorporating an irregular heartbeat indicator to estimate whether atrial fibrillation is present) and getting the patient or a relative to gently press on the lower leg (*digital pressure*) to produce an indentation (*pitting*) that is indicative of edema.

These examinations appeared to be very straightforward when the clinician and patient were colocated. However, in the remote setting, the same physical examinations presented specific challenges: (1) adequate design of instructions to guide video examinations (nurses had to explain tasks using lay language and check that instructions were being followed), (2) accommodation of the patient’s desire for autonomy (on the part of nurses and relatives) in the light of opportunities for involvement in their own physical assessment, and (3) conducting a physical examination while simultaneously making it visible to the nurse (patients and relatives needed adequate technological knowledge to operate a device and make the examination visible to the nurse as well as basic biomedical knowledge to follow nurses’ instructions). We have presented a detailed analysis of these challenges and the strategies used to attempt to overcome them in a separate paper [77].

Heart failure patients tend to be elderly, and many have poor exercise tolerance, mobility problems, or comorbidities, making attendance at the clinic potentially difficult. For the 9 patients in our sample attending the consultation in person, physical examinations were all successfully conducted by the clinician with minimal assistance from attending carers. For the 7 consulting by video, all participants (clinicians, patients, and, sometimes, relatives) had to employ novel communication strategies to collaboratively negotiate the challenges of achieving a satisfactory physical examination in the remote setting. Participants relied on a range of unspoken assumptions about the technology and their respective knowledge and understanding of medical procedures and the meaning of measurements. Clinicians and patients ran into unforeseen problems, either with the procedure (eg, the patient put a blood pressure meter incorrectly) and/or the technical process (eg, video quality prevented full visual assessment). Although complex, in 5 consultations, these problems were resolved with patients and/or their relatives asking the clinician for feedback or instructions and working with them to successfully complete the examination.

**Figure 4:**
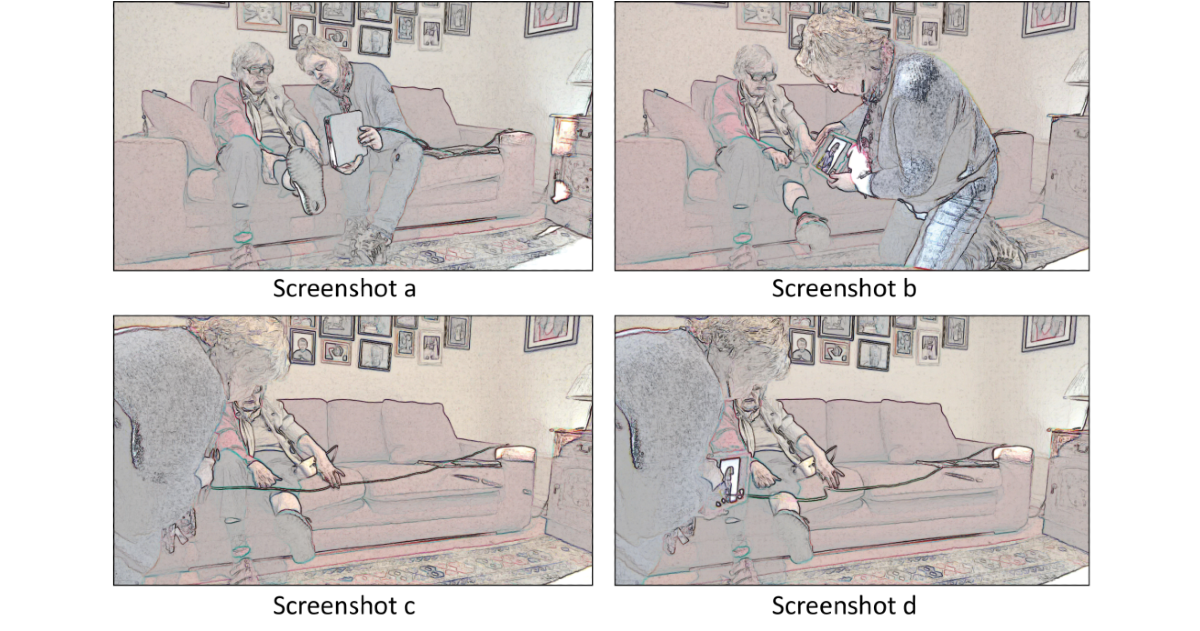
Heart failure patient and relative attempting examination for edema.

There were 2 consultations in which it was not possible to complete an attempted physical examination. In both instances, this was because the patient or relative was not able to hold the phone or tablet and test for edema at the same time. Take the example in Figure 4, of a heart failure patient and her daughter, in which they have been instructed by the clinician to examine the patient’s right leg for edema; the daughter attempts to aim the camera at the patient’s leg while at the same time trying to monitor the screen, as can be seen in the top 2 screenshots. This does not work adequately, and the nurse is not able to get a view of the patient. When she finally—after minutes of moving around—manages to aim the camera at the patient’s leg (screenshot c, Figure 4), she is instructed by the nurse to press into the patient’s leg. She lets go of the tablet with one hand and loses control (screenshot d, Figure 4), the result being that the camera is no longer aimed at the patient’s leg. The nurse thus cannot assess if the patient has edema, and following this failed attempt, they agree to stop the examination. It is also worth noting that the patient end of the examination takes place in a living room that was not designed for clinical examinations. The sofa is much lower than a seat in a clinic, there is no examination couch, and the lighting in the home and on the tablet is not designed to provide the level of illumination considered standard for a clinical examination.

The 86-year-old patient shown in Figure 4 knows the nurse well and has seen her before in the clinic and at home. Having recently lost her husband, she had not been well and was admitted to the hospital with pneumonia. She deteriorated, resulting in close management by her GP and the specialist heart failure nurse. This story of acute deterioration in an older patient with multimorbidity is typical of the 16 patients with heart failure in our sample. Similar to the patient in Figure 4, those consulting via video were also doing so from a space (typically a living room) that was not designed for clinical examinations in which chairs were often too low, there was no examination couch, lighting was limited to standard bulbs, and there were often personal objects (eg, lamps or ornaments) that might impede movement or be knocked over. Hence, although 5 of the 7 remote examinations were completed, many found the process physically, practically, and technically challenging. Most were familiar with the technology from a clinic visit but had not necessarily practiced either in this kind of assessment or in using multiple devices to aid the process, especially in an environment that had not been designed with clinical assessment in mind. Unlike in face-to-face consultations, success was often dependent on the type of technology (smartphone, tablet, or laptop), the presence of a third party who could assist the patient, the patient’s mobility, and the technological competence of the participants. Participants often reflected that it might be easier to perform a remote physical examination the second time as they were now aware of the problems they might encounter.

## Discussion

### Principal Findings

Our study has shown that, at least for this group of patients and clinicians, interaction in the majority of video consultations is effective. Observation and recording of video consultations, combined with theoretical sensitivity to the ways in which technology mediates communication in clinical consultations, has enabled us to do the following. First, we have shown that when participants experience technical or operational issues in video consultations (eg, when experiencing a problem connecting due to latency), they generally find a way to negotiate these, falling back on tried and tested rules of communication transferred from face-to-face situations. Familiarity with technology helps. Second, in a small proportion of consultations, technical interruptions before or during consultations may require workarounds such as using a standard telephone line to overcome lack of audio, and, in a few cases, it may threaten the quality of communication and clinical safety. Third, we have shown that in the minority of cases where significant latency occurs, clinicians and patients do not always hear the same thing at the same time. Finally, we have revealed that video consultations necessitate patients (and potentially carers too) to take an active role in understanding and manipulating the technology (eg, to change what the other party can see) and their surroundings (eg, adjusting lighting). For those consultations involving a remote physical examination, this involves a complex process of giving and receiving instructions and ensuring visual presence.

### Strengths and Limitations

Careful analysis of verbal and nonverbal interaction, combined with a focus on social context, has enabled a robust understanding of the role played by language and social interaction in video consultations. Such detailed analysis takes time. Although we were fortunate to have data from 2 separate and fairly large qualitative studies, these comprised a relatively limited range of consultations in only 4 clinical conditions. Only 1 of these, heart failure, involved remote physical examinations.

CA is an established methodological technique for studying social interaction that allowed us to systematically identify challenges and to then generate and analyze relevant collections across our dataset. This is a key strength of our study. When the original studies were designed, we did not set out to examine 1 of these challenges—latency. This meant that the dataset we analyzed did not allow us to determine the precise timings of all instances of latency. When selecting data to analyze the impact of latency on conversational flow we therefore focused on cases where both participants talked in overlap following a point where turn transition could have taken place (eg, after a question) [54]. Future studies of latency in video consultations should ensure a comparison of recordings from both *ends* at every point.

Our dataset included video and audio recordings of consultations using Skype and FaceTime because these were the platforms being used at the time. Neither of these platforms was specifically designed for clinical consultations, and it may be that next-generation video consultation software (eg, Attend Anywhere) or more advanced peripheral technologies, such as noise-canceling microphones, may produce less in the way of breakdowns in conversations. We recommend that any future published studies of video interactions include details of the specific technologies used.

### Comparison With Other Studies

To our knowledge, our study is one of the first to apply linguistic ethnographic approaches to the study of video-mediated consultations in health care. As such, it represents a significant addition to the existing literature, which has been dominated by trials and focused on issues of feasibility and acceptability and has also included some conventional CA without linguistic ethnography [44,46]. It extends our previous research on this dataset of video-mediated consultations, which include a multilevel analysis of the policy, organizational, and interactional aspects (using the Roter interaction analysis system) [9,47], and an in-depth analysis of physical examinations via video [77].

Our research confirms findings from our own and other studies that there are interactional differences between video and face-to-face consultations and potential for collaborative decision making via video [30,43,44,48,49,78]. Our study also corroborates that people tolerate some silence in conversation but work to minimize it [57] and that delays of 0.5 to 1 second can cause significant problems [52,53]. We have added to this, providing a detailed account of the frequency and duration of breakdown and latency issues specific to video consultations, showing that, in line with studies of how overlapping talk is negotiated in face-to-face conversations [79,80], participants have strategies for dealing with these problems.

Previous work has shown that speakers typically rely on what phenomenologists refer to as *reciprocity of perspectives* [81], meaning that both parties assume that the other hears and sees what they hear and see. Any differences, as far as speakers can be aware, are assumed to be irrelevant until proven otherwise [82]. Our study is the first to explore the relevance of this in video consultations in clinical settings, with 29 cases identified in which participants acted as if there were no delays and were not aware that there was a delay. This meant not only that it took them longer to solve the problem of overlapping talk but also that when they used conventional strategies for solving overlapping talk, this could lead to new problems (Figure 3). This has potentially significant implications for quality and safety, for instance, when communicating medication dosage.

### Meaning of the Study

Our findings suggest that care is needed on the part of health care providers, commissioners, and policy makers in rolling out this new service model. Our own study and that of others has shown that video consultations appear to be largely safe and effective (albeit in a small sample of clinics and clinicians, and with patients identified by clinicians as *suitable* for a video consultation) [8,9,83], follow the conventional format of a face-to-face consultation, and any interactional challenges tend to be overcome. However, there is also a potential for significant problems, for instance, around miscommunication of dose or diagnosis, or misunderstandings because of technological breakdown.

Until recently, video consultations have tended to be an optional extra for many working in health services. The specter of COVID-19 has rapidly changed the landscape [84]. Clinicians and patients face a high risk of infection if they consult face-to-face, meaning that video consultations have significant advantages, both in cases where patients or clinicians are self-isolating because of symptoms of COVID-19 and where patients with other conditions are being seen remotely in an effort to reduce infection risks. In short, with a rapidly spreading disease, the pressure is on to reduce the number of people consulting face-to-face. Rolling out such a service at speed is, in some ways, similar to what we have studied in that many consultations are likely to involve dealing with routine, nonacute issues in a range of conditions with already known patients.

Before the COVID-19 pandemic, resources to support video consulting tended to be ad hoc and limited. In most cases, clinicians and patients were conducting video consultations without guidance for what works for which patients, clinical condition, or stage of diagnosis. That remains the case at the time of publication with, if anything, increased uncertainty about how video consultations can best be used as workflows are rapidly reorganized during the COVID-19 crisis. This lack of guidance means that clinicians and patients can run into unforeseen problems and communicative challenges that they may not know how to negotiate or easily resolve. Some problems potentially go unnoticed (eg, inadequate lighting misrepresents the patient’s condition in a remote physical examination), which poses potential risks to the patient.

Guided by the findings from this and previous studies and in collaboration with patients (see above), we have developed a suite of freely available resources for patients and clinicians (available online [85] and summarized in Multimedia Appendix 4). This includes guidance on setting up and running video consultations for patients and clinicians, sets of frequently asked questions, downloadable leaflets and posters, and an animation. Our original intention, before the COVID-19 epidemic, was to provide a generic resource that could support patients or clinicians in setting up and running a video consultation. This remains the case. However, guided by emerging evidence, we have adapted guidance to ensure accessibility and relevance for those using video calling during and beyond the COVID-19 pandemic.

### Unanswered Questions and Future Research

Communication in video consultations is under-researched. Further qualitative work is needed to examine interactional issues across a broader range of settings, conditions, and populations to inform the development of video consultations and to support patients and clinicians who choose to use them. This includes other kinds of remote physical examination for other conditions and with other types of patients (including those like older people or those with frailty who typically need more health care consultations but have limited experience of video technology).

Questions remain about the extent of latency, its often subtle effects [80], and the implications of how overlapping talk is understood differently by each participant. Further work is needed to consider a wider range of overlapping talk and to gain detailed understanding of how latency affects conversational flow and may cause misunderstandings in video consultations.

There have been significant advancements in technology in recent years, enabling both standalone platforms (eg, Skype) and those dedicated to video consultations (eg, Attend Anywhere). Future studies should examine what, if any, technical and interactional benefits the latter bring, including improved peripherals (eg, better screens or webcams) and use of companion devices (ie, multiple devices in combination).

### Conclusions

Video consultations are interactionally different from face-to-face consultations. The use of video technology has the potential to change the way in which patients and clinicians interact, particularly when problems with audio or video interrupt the usual flow of interaction or where a physical examination is required. The use of evidence-based guidance, combined with training and support, can help clinicians and patients to more quickly identify and work through technical problems and avoid the potential for significant misunderstanding. Such resources are likely to be essential if video consultations are to be delivered at scale.

## Appendix

Multimedia Appendix 1 Screenshot of Transana workspace.

Multimedia Appendix 2 Transcriptions conventions for conversation analysis.

Multimedia Appendix 3 Full transcriptions for Figures 1-3, using conversation analysis conventions.

Multimedia Appendix 4 Summary guidance on video consultations for clinicians and patients.

## Abbreviations

BRC: Biomedical Research Centre
CA: conversation analysis
COVID-19: coronavirus disease 2019
GP: general practitioner
NHS: National Health Service
NIHR: National Institute for Health Research
OTQS: Oxford Telehealth Qualitative Study
PAG: patient advisory group
QuARC: Qualitative Analysis of Remote Consultations
VOCAL: Virtual Online Consultations-Advantages and Limitations
