# Supplementary figures and images for: Video Consultations Between Patients and Clinicians in Diabetes, Cancer, and Heart Failure Services: Linguistic Ethnographic Study of Video-Mediated Interaction
Source: J Med Internet Res. 2020 May 11;22(5):e18378. doi: 10.2196/18378 (PMC7248806; doi:10.2196/18378)

### Screenshot of Transana workspace

| 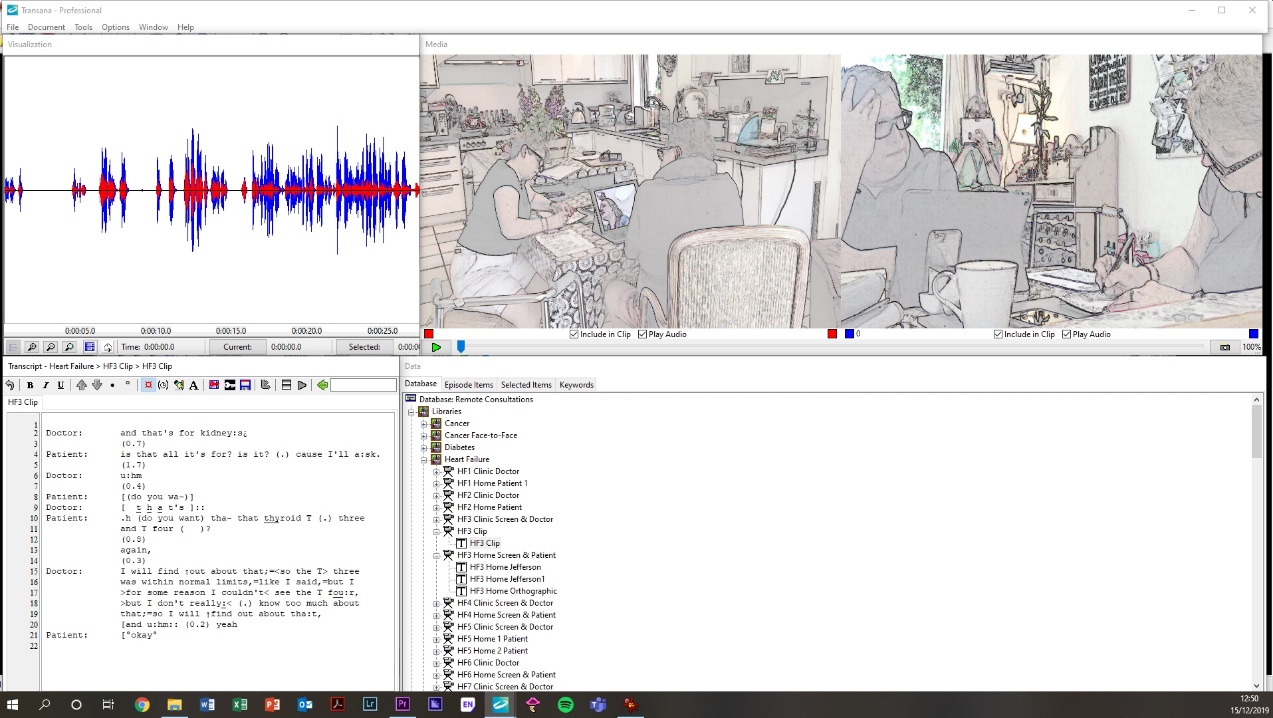 |
| --- |

Supplement: Multimedia Appendix 1 [file jmir_v22i5e18378_app1.docx]
