# Supplementary material for: Video Consultations Between Patients and Clinicians in Diabetes, Cancer, and Heart Failure Services: Linguistic Ethnographic Study of Video-Mediated Interaction
Source: J Med Internet Res. 2020 May 11;22(5):e18378. doi: 10.2196/18378 (PMC7248806; doi:10.2196/18378)
Supplement: Multimedia Appendix 2 [file jmir_v22i5e18378_app2.docx]

### **Multimedia Appendix 2: Transcription conventions used (in combination) in the QuARC study**

#### Jeffersonian Transcription Conventions

| Symbol | Meaning |
| --- | --- |
| (1.0) | Numbers between parentheses represent seconds of silence. |
| (.) | A silence of less than 200ms |
| turn1=  =turn2 | There is no silence between the two turns. |
| tal[k  [talk | Overlapping talk. The left square bracket marks the point of overlap onset. |
| talk]  tal]k | Overlapping talk. The right square bracket marks the point where overlap ends. |
| talk. | Prosodic completion with a boundary pitch that falls to low in the speaker’s range. |
| talk, | Prosodic completion with a boundary pitch that rises to the middle of the speaker’s range. |
| talk? | Prosodic completion with a boundary pitch that rises to high in the speaker’s range. |
| talk; | Prosodic completion with a boundary pitch that falls to the middle of the speaker’s range. |
| talk_ | Prosodic completion with a flat boundary pitch. |
| ↑ | Upstep in the speaker’s pitch that lasts no longer than one syllable. |
| ↓ | Downstep in the speaker’s pitch that lasts no longer than one syllable. |
| stre::tch | Vowel or consonant is held longer. |
| stress | Talk is pronounced with audible stress or emphasis. |
| pi:tch | An underlined vowel followed by a colon that is not underlined  signifies a pitch that rises and falls during the production of  the vowel. |
| LOUD | Capitals signify relatively loud speech. |
| °soft° | Degree signs signify relatively soft speech. |
| ^high^ | Carets signify speech high in the speaker’s pitch range. |
| >talk< | Contracted or relatively fast speech |
| tal- | A hyphen signifies a cut-off in mid-production, typically audible as a glottal stop. |
| .hh | Audible inbreath. Each *h* denotes about 200ms. |
| £talk£ | The speaker is audibly smiling while speaking. |
| ((sniffs)) | Comment, typically a characterization of a sound that cannot be represented otherwise. |
| ((carer)) | Anonymization; name was removed and replaced by role. |
| ( ) | Inaudible talk |
| (talk) | Not clear what speaker said, only an attempt could be made at transcription. |
| (talk/talk) | Not clear what the speaker said; slash separates two ways the data could be heard. |

##### Table 3: Mondada Transcription Conventions

| Symbol | Meaning |
| --- | --- |
| * *  + +  $ $ | Gestures and descriptions of embodied actions are delimited between two identical symbols (one symbol per participant) and are synchronized with correspondent stretches of talk |
| *--->  --->* | The action described continues across subsequent lines,  until the same symbol is reached |
| >> | The action described begins before the excerpt’s beginning. |
| --->> | The action described continues after the excerpt’s end |
| ….. | Action’s preparation |
| ---- | Action’s apex is reached and maintained |
| ,,,,, | Action’s retraction |
| nur | Participant doing the embodied action is identified when they are not the speaker |
| fig  # | The exact moment at which a screenshot has been taken is indicated,  with a pound symbol showing its position within turn at talk |
