# Supplementary material for: Video Consultations Between Patients and Clinicians in Diabetes, Cancer, and Heart Failure Services: Linguistic Ethnographic Study of Video-Mediated Interaction
Source: J Med Internet Res. 2020 May 11;22(5):e18378. doi: 10.2196/18378 (PMC7248806; doi:10.2196/18378)
Supplement: Multimedia Appendix 3 [file jmir_v22i5e18378_app3.docx]

| **Table 1: Transcript of figure 1 with full Conversation Analysis conventions** | |
| --- | --- |
| 01 Da: °o::h. did I just answer  02 wrong°?=  03 Dr: =(no)  04 (3.0)  05 Ns: °( )°  06 Da: [°there's:: the doctor mum.  07 Dr: °( )°  08 (3.9)  09 Dr: helLO:?  10 (.)  11 Da: hi::,  12 (0.4)  13 Ns: hi:. [(we) can't see you¿  14 Dr: [HI.  15 (0.4)  16 Dr: [we can hear you but we]  17 Da: [ you can't see us:?]  18 Dr: can't see you;=can you  19 see us?  20 (0.2)  21 Da: I can see you:?  22 hold o:n,  23 (1.4)  24 Dr: °you need to° (.) turn your  25 camera on?=  26 Ns: =(oh)  27 (.)  28 Da: can you see n[ow:?  29 Dr: [ye:s yes yes,  30 hello we can see you now? | 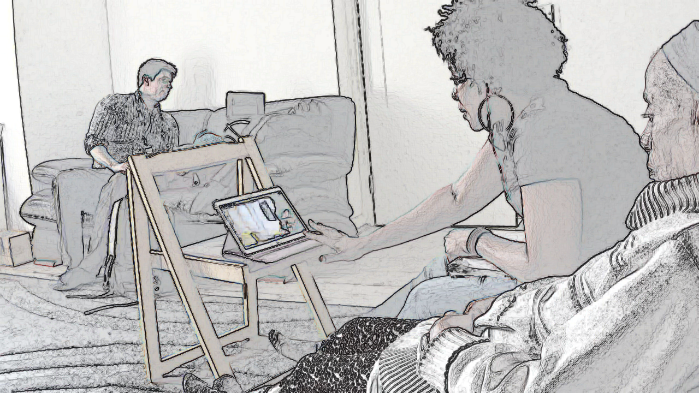 |

| **Table 2: Transcript of figure 2 with full Conversation Analysis conventions** | |
| --- | --- |
| 01 Dr: one suggestion,  02 (0.9)  03 Dr: you're taking the ten thousand  04 capsules, [isn't it.  05 Pt: [yes.  06 (.)  07 Dr: they are also available at twenty-five  08 thousand.  09 (2.3)  10 so: (0.4) so you ↑could take (0.2) <just  11 one capsule with light (.) meals>,  12 and then two: [or three for big meal:s.  13 Pt: [a:h.  14 I see:.  15 (0.4)  16 u:hm at the ( )¿ they do ↑not uh:m  17 make (0.2) five ↑(thousand) ones.  18 [u::h (you) can only: get ten thousand  19 Dr: [hm:  20 Pt: ones.=( ) have to:- yes:.  21 I (could)- (.) I could (lower) the dose.  22 (.)  23 [yeah  24 Dr: [you c- you could  25 ↑ask your GP the next time you:  26 [get the (creon),  27 Pt: [mm hm, | Start of audio distortion  End of distortion |

| **Table 3: Transcript of figure 3 with full Conversation Analysis conventions** | | |
| --- | --- | --- |
|  | What the patient heard  (i.e. recorded at the patient’s end) | What the nurse heard  (i.e. recorded at the nurse’s end) |
| 01  02  03  04  05  06  07  08  09  10  11  12  13  14  15  16  17  18  19  20  21  22  23  24  25  26  27  28 | Pt: uhm I told you about playing  croquet? as you know I  played croquet last year;  .h[hh this year .h I'm  Ns: [yeah,  Pt: struggling to complete  one round.  (0.5)  without having to sit d[own.  Ns: [are  you really;  Pt: yea:h.  (0.5)  u:hm I'm gonna keep on at  it, I'[ve got a-]  Ns: [and is i]t a pos-  (1.0)  Pt: sorry,  (1.5)  .hh[h  Ns: [yeah go on¿  Pt: I:'ve g[ot- ]  Ns: [>y'r gonna] keep  on<,  (0.4)  Pt: yeah I've got a .hh  competition at ((place  name)) tomorrow | Pt: uhm I told you about playing  croquet? as you know I  [played croquet last year;  Ns: [yeah,  Pt: .hhh this year I'm  struggling to complete  one round.  Ns: are you really;  Pt: without having to sit down.  (0.6)  Pt: [yeah.  Ns: [(hm)  (0.4)  and is i[t a p o s-]  Pt: [I'm gonna kee]p  on at it, I've-  (1.9)  Pt: sorry,  Ns: g- yeah go on¿  (0.5)  Ns: y'r gonna keep on,  (0.2)  Pt: I've got- (1.0) yeah I've got  a .hh competition at ((place  name)) tomorrow. |
