# Supplementary material for: Video Consultations Between Patients and Clinicians in Diabetes, Cancer, and Heart Failure Services: Linguistic Ethnographic Study of Video-Mediated Interaction
Source: J Med Internet Res. 2020 May 11;22(5):e18378. doi: 10.2196/18378 (PMC7248806; doi:10.2196/18378)
Supplement: Multimedia Appendix 4 [file jmir_v22i5e18378_app4.docx]

**MULTI MEDIA APPENDIX 4:
SUMMARY GUIDANCE ON VIDEO CONSULTATIONS FOR CLINICIANS AND PATIENTS**

Barts Health NHS Trust and the University of Oxford have been working in collaboration, along with Design Science and others, to develop a suite of resources for patients and clinicians on ‘how to do a video consultation’.

Generic resources were due to be published at the end of March. In light of the Covid-19 crisis we sped up this process, and made changes to the guidance to ensure relevance in the context of the broader health system response to coronavirus, which involves significant and rapid expansion of the use of video consultations.

The following patient and clinician resources will be free available to download and use, via University of Oxford (<https://www.phc.ox.ac.uk/research/resources/video-consulting-in-the-nhs>) and Design Science (<https://design-science.org.uk/nhs-video-consulting/>):

1. 2 minute video guide for patients
2. Patient ‘quick guide’ on video consulting
3. Patient FAQs
4. Clinician 'quick guide’ on video consulting
5. Clinician FAQs
6. Video consultation information for NHS Trusts
7. Video consultation information for GPs
8. GP A4 printable guidelines

We have included DRAFT flowcharts of guidance for patients and clinicians below to provide readers with an indication of the content of resources. Full and final resources are available via the websites above, having been designed and illustrated with support from Design Science, an applied design company that has helped us to bring the evidence on doing video consultations to life and ensure high quality, accessible information.

1. **Flowchart for Clinicians, summarising the process of setting up and doing a video consultation***

* Detailed guidance at <https://www.phc.ox.ac.uk/research/resources/video-consulting-in-the-nhs>

1. **Flowchart for Patients, summarising the process of setting up and doing a video consultation***

* Detailed guidance at <https://www.phc.ox.ac.uk/research/resources/video-consulting-in-the-nhs>
